# Supplementary material for: Angelica sinensis polysaccharide as potential protectants against recurrent spontaneous abortion: focus on autophagy regulation
Source: Front Med (Lausanne). 2025 Jan 15;12:1522503. doi: 10.3389/fmed.2025.1522503 (PMC11774876; doi:10.3389/fmed.2025.1522503)
Supplement: Supplementary material S1 — The main instruments used during the LC-MS process, along with their models/specifications and manufacturers. [file Supplementary_file_1.zip › Metabolomics sequencing data FC1.2/非靶向代谢-LCMS-EMDB-1.0.0-分析报告.html]

  非靶向代谢-LCMS-EMDB 项目结题报告
Report


# 非靶向代谢-LCMS-EMDB 项目结题报告

**项目信息**


项目类别
:   非靶向代谢-LCMS-EMDB

项目编号
:   DLM202213432

客户名称
:   谈媛

联系人名称
:   王同帅

样本类型
:   小鼠组织

映射物种
:   mmu

执行编号
:   LM0141

---


#### 感谢您选择欧易生物！

**关于网页报告使用有以下几点提示:**

1. **报告正文**中的**蓝色字体**均可以**点击**快速导引到感兴趣的章节, 便于您快速查看相应内容。
2. **报告正文**中的**图片**均可以点击后进行**放大查看**, 且图片点击后可以包含更多的细节信息, 比如左上角会显示具体的**图片数量**, 右上角有相关的**图片工具**, **键盘上的左右方向键/鼠标滚轮**可以对图片进行**切换**。
3. **报告正文**中**每个区域**中图片的数量**最多**只显示2张, 更多的图片可以在点击之后在弹出界面利用**键盘方向键/鼠标滚轮**查看。
4. 展示的表格均可以在各列**表头**进行**升序或者降序显示**, 当表格多于20行的时候, 表格会嵌入到网页之中, 可以利用滚动栏进行调整设置, 同时会在表格上方增加搜索设置, 便于快速查询表格信息。
5. 在报告中浏览, 需要返回顶部的时候, 可以使用网页**右下角的白色箭头标识**快速返回顶部。
6. 本提示可以点击右上方的不再显示, 则后续打开网页均不会显示本提示。

### 1.背景信息

---

- 1.背景信息
- 2.数据分析
  - 2.1.基峰图
  - 2.2.质量控制
    - 2.2.1.QC样本质控
      - 2.2.1.1.QC样本主成分分析
      - 2.2.1.2.代谢物强度分布评估
      - 2.2.1.3.QC样本层次聚类
  - 2.3.数据预处理
    - 2.3.1.定性定量结果
    - 2.3.2.Progenesis QI v3.0的定性分析
    - 2.3.3.代谢物分类
- 3.差异比较分析
  - 3.1.多元统计分析
    - 3.1.1.主成分分析(PCA)
    - 3.1.2.偏最小二乘-判别分析(PLS-DA)
    - 3.1.3.正交偏最小二乘方-判别分析(OPLS-DA)
    - 3.1.4.Permutation
    - 3.1.5.Loading\_plot
    - 3.1.6.Splot
  - 3.2.单变量统计分析
  - 3.3.差异代谢物筛选
    - 3.3.1.差异代谢物聚类热图
    - 3.3.2.差异代谢物火山图
    - 3.3.3.差异代谢物箱型图
    - 3.3.4.Z-Score标准化
  - 3.4.相关性分析
    - 3.4.1.相关性图
    - 3.4.2.相关性网络图
  - 3.5.代谢通路富集分析
    - 3.5.1.通路富集分析气泡图
    - 3.5.2.代谢通路图
- 4.项目结论
- 5.常见问题
- 6.研究案例
  - 6.1.代谢组学揭示土壤中纳米材料暴露改变土壤根际代谢物特征和玉米代谢途径
  - 6.2.木香挥发油治疗裸鼠MCF-7异源乳腺癌移植瘤模型的代谢组学研究
- 7.公司简介
  - 7.1.公司仪器
  - 7.2.公司服务
- 8.申明

copyright© 2020 上海欧易生物科技有限公司

地址:上海市闵行区浦江镇联航路1188号25号楼

电话：4008890037
